# Human PrimPol activity is enhanced by RPA

**DOI:** 10.1038/s41598-017-00958-3

**Published:** 2017-04-10

**Authors:** María I. Martínez-Jiménez, Antonio Lahera, Luis Blanco

**Affiliations:** Centro de Biología Molecular Severo Ochoa (CSIC-UAM), c/Nicolás Cabrera 1, 28049 Cantoblanco, Madrid Spain

## Abstract

Human PrimPol is a primase belonging to the AEP superfamily with the unique ability to synthesize DNA primers *de novo*, and a non-processive DNA polymerase able to bypass certain DNA lesions. PrimPol facilitates both mitochondrial and nuclear replication fork progression either acting as a conventional TLS polymerase, or repriming downstream of blocking lesions. *In vivo* assays have shown that PrimPol is rapidly recruited to sites of DNA damage by interaction with the human replication protein A (RPA). In agreement with previous findings, we show here that the higher affinity of RPA for ssDNA inhibits PrimPol activities in short ssDNA templates. In contrast, once the amount of ssDNA increases up to a length in which both proteins can simultaneously bind ssDNA, as expected during replicative stress conditions, PrimPol and RPA functionally interact, and their binding capacities are mutually enhanced. When using M13 ssDNA as template, RPA stimulated both the primase and polymerase activities of PrimPol, either alone or in synergy with Polε. These new findings supports the existence of a functional PrimPol/RPA association that allows repriming at the exposed ssDNA regions formed in the leading strand upon replicase stalling.

## Introduction

DNA polymerases are the enzymes responsible for making and repairing the DNA to ensure cell viability. Polymerases cannot initiate DNA synthesis, but require a 3′ OH provided by a pre-existing primer that will be elongated with incoming nucleotides. In many cases, initiation of DNA replication is performed by specialized RNA polymerases or “primases” which synthesize RNA primers that are transferred to a DNA polymerase. PrimPols belong to the archaeo-eukaryotic primase superfamily, and are unique, as they display not just RNA primase activity, but also DNA primase and DNA-dependent DNA polymerase activities, which share the same active site of the enzyme. Thus, PrimPol provides the replicative process with a new alternative to start DNA synthesis: a DNA primer. This combination of biochemical properties has been described in PrimPols from plasmids^1, 2^, bacteria^3^, archaea^4, 5^ and humans^6–8^.

Human PrimPol was shown to be present in both nucleus and mitochondria, and its two DNA synthesizing activities, DNA primase and DNA polymerase, were proposed to contribute to DNA damage tolerance during DNA replication^7^. Compelling evidence demonstrates that the DNA primase activity of human PrimPol facilitates DNA replication by repriming DNA synthesis beyond lesions, bulky structures or any circumstance that block elongation, leaving behind an unreplicated gap to be repaired post-replicatively^9–11^. Accordingly, those DNA primers synthesized by human PrimPol can be efficiently elongated by the replicative polymerases Polγ and Polε^7^. Moreover PrimPol can also act as a translesion synthesis (TLS) DNA polymerase, which is able to tolerate readable lesions (as 8oxoG)^7, 12^, or to avoid others as AP sites and 6-4PP by reannealing the primer terminus to a position ahead of the lesion^9, 13^. Such TLS abilities of human PrimPol are concomitant with a distributive pattern of DNA polymerization, lack of proofreading activity, but having a moderate insertion fidelity like other TLS polymerases^13^.

Human PrimPol possesses two distinct domains: (1) an N-terminal AEP polymerization domain containing the three signature motifs required for catalysis; (2) a UL52-like zinc finger (Zn-finger) domain which is necessary for the primase activity (probably to stabilize the nascent primer)^9, 14^. Recently, the 3D structure of the AEP domain of human PrimPol (aa 1-348) has been solved in complex with both DNA and incoming dNTP^15^ (PDBid: 5L2X). Co-immunoprecipitation assays have demonstrated that the most carboxy-terminal region (after the Zn-finger domain) of human PrimPol mediates the interaction with the large subunit of replication protein A (RPA), the human single-stranded DNA binding protein^6^. Moreover, Guilliam *et al*.^16^ have shown that the N-terminal region of RPA70 is the responsible for the interaction with PrimPol^16^.

*In vivo* assays showed that PrimPol is rapidly recruited to sites of DNA damage induced by UV irradiation, independently of the checkpoint response^9^. That recruitment was shown to require the C-terminal region of PrimPol that contains a putative RPA interaction site^6^. In fact, expression of a PrimPol variant defective in the carboxy-terminal region was incapable of preventing the appearance of γH2AX *foci*, and suppressing the elevated HU sensitivity when endogenous PrimPol was silenced^6^.

Strikingly, although PrimPol recruitment to stalled forks seems to be mediated by RPA, it was proposed that RPA elicits an inhibitory effect on PrimPol activities in order to limit error-prone synthesis^16^. Such a paradox is explored here in this paper, by studying the functional interaction of these two proteins, and how the length of the template defines either a stimulatory or inhibitory effect of RPA on PrimPol catalytic activities.

## Materials and Methods

### DNA, oligonucleotides and nucleotides

M13 ssDNA was supplied by New England Biolabs (Ipswich, MA, USA). DNA oligonucleotides were synthesized by Sigma Aldrich (St Louis, Mo, USA). Unlabeled ultrapure dNTPs were supplied by GE (Fairfield, CT, USA). Radiolabeled nucleotides [γ-^32^P]ATP and [α-^32^P]dGTP (250 µCi; 3000 Ci/mmol) were obtained from Perkin Elmer (Waltham, MA, USA). T4 polynucleotide kinase used for 5′oligonucleotide labeling, was supplied by New England Biolabs (Ipswich, MA, USA).

### Purification of PrimPol and RPA

PrimPol wild type was overexpressed and purified as previously described^7^. RPA expression vector was a gift from Barbara van Loon (NTNU, Norway). RPA was overexpressed and purified as described^17^ (see Supplemental Fig. 1). Polε was a gift from Zachary F. Pursell (Tulane University, USA).

### Polymerase assay on specific template/primer molecules

Short template/primer: Oligonucleotide 16-mer (5′-CACTGACTGTATGATG-3′), used as primer, was labeled with PNK and [γ-^32^P]ATP as indicated by the manufacturer; the labeled primer was hybridized to a 30-mer oligonucleotide template (5′-CTCGTCAGCATCXXCATCATACAGTCAGTG-3′) where XX is either TT (undamaged template) or 6-4 photoproduct thymine dimer site (6-4PP), kindly provided by S. Iwai (Osaka University, Japan). Long template/primer: oligonucleotide 16-mer (5′-GAACCTGCAGGTGGGC-3′), used as a primer, was labeled as described before and hybridized to a 65-mer oligonucleotide (5′-CGCTGCCGAATTCTACCACGCTACTAGGG TGCCTTGCTAGGACATCTTTGCCCACCTGCAGGTTC-3′), used as template. Reactions (in 20 μL) were carried out in a buffer containing: 50 mM Tris-HCl [pH 7.5], 50 mM NaCl, 1 mM MnCl_2_, 1 mM DTT, 2.5% glycerol, 0.1 mg/ml BSA, 2.5 nM template/primer DNA and 100 µM dNTPs in the presence of PrimPol (200 nM). When indicated, RPA was added in increasing concentration (12.5, 50, 200 nM) immediately after PrimPol addition. After incubation during 30 min at 30 °C, reactions were stopped by adding 8 μL of formamide loading buffer (95% formamide, 20 mM EDTA, 0.1% xylene-cyanol, and 0.1% bromophenol blue), and loaded onto 20% polyacrylamide sequencing gels containing 8 M urea. Following denaturing electrophoresis, autoradiography was used to detect primer extension.

### Polymerase assay on primed-M13 ssDNA

Reactions (in 20 μL) were carried out in a buffer containing: 50 mM Tris-HCl [pH 7.5], 50 mM NaCl, 100 μM MnCl_2_, 5 mM MgCl_2_, 1 mM DTT, 2.5% glycerol, 0.1 mg/ml BSA, 5′[γ-^32^P]-labeled primer (5′-GTTTTCCAGTCACGAC-3′) annealed to M13 ssDNA (2.5 nM), 100 µM dNTPs, PrimPol (100 nM) and RPA when indicated (20, 100 and 500 nM), were incubated for 10 min at 37 °C. When indicated, PrimPol and RPA were pre-incubated 10 min at RT before template/primer addition. Reactions were stopped by adding 8 μL of loading buffer, then heated 5 min at 80 °C and analyzed in a 15% polyacrylamide sequencing gels containing 8 M urea.

### Primase assays on specific oligonucleotide templates

Two different size versions of the “GTCC” oligonucleotide (29-mer 5′-T_15_CCTGT_10_-3′ or 60-mer 5′-T_36_CCTGT_20_-3′), which contain a putative herpes virus priming initiation site^18^, were used as favorite templates. The reaction (20 μL) was carried out in a buffer containing: 50 mM Tris-HCl [pH 7.5], 50 mM NaCl, 1 mM MnCl_2_, 1 mM dithiothreitol (DTT), 2.5% glycerol, 0.1 mg/ml (BSA), [γ-^32^P]ATP and dGTP (1, 10, 100 µM) in the presence of PrimPol (100 nM) and the indicated “GTCC” oligonucleotide (1 µM). When indicated, RPA was added in increasing concentration (12.5, 50, 200 nM) to the reaction that contains 100 nM PrimPol and 100 µM dGTP. After 60 min at 30 °C, reactions were stopped by the addition of formamide loading buffer. Products were loaded in 8 M urea-containing 20% polyacrylamide sequencing gel. After electrophoresis, *de novo* synthesized polynucleotides were detected by autoradiography. Alternatively, a 65-mer oligonucleotide (5′-CGCTGCCGAATTCTACCACGCTACTAGGGTCCTTGCTAGGACATCTTTGCCCACCTGCAGGTTC-3′) was used as an unspecific template. The reaction (20 μL) was carried out in the buffer described above, but using [α-^32^P]dGTP (16 nM), dNTPs (100 µM) as indicated, 65-mer oligo (1 µM), PrimPol (100 nM), either in the absence or presence of RPA (200 nM). The reaction was incubated and analyzed as described above.

### Electrophoretic mobility shift assays (EMSAs)

Reactions (in 20 μL) were carried out in a buffer containing: 30 mM Hepes, 0.5% Inositol, 1 mM DTT, 2.5% glycerol, 0.1 mg/ml BSA, 5 mM MgCl_2_, 100 µM MnCl_2_, 50 mM NaCl, 15 mM KCl and 2.5 nM ssDNA 65-mer (5′-CGCTGCCGAATTCTACCACGCTACTAGGGTGCC TTGCTAGGACATCTTTGCCCACCTGCAGGTTC-3′) and the indicated concentration of PrimPol and/or RPA. The reaction was first incubated 5 min at RT without DNA and later 20 min at 25 °C. Reactions were loaded in a 6% polyacrylamide gel with EMSA loading buffer (40% glycerol, bromophenol blue and xylen-cyanol). After electrophoresis at 4 °C, the gel was dried and analyzed by autoradiography.

### Primase-polymerase combined assays

Reactions (in 20 μL) contained: 50 mM Tris-HCl [pH 7.5], 50 mM NaCl, 15 mM KCl, 1 mM DTT, 2.5% glycerol, 0.1 mg/ml BSA, 2 nM M13-ssDNA template, 100 µM GTP, 100 µM dATP/dCTP/dTTP, 16 nM [α-^32^P]dGTP, 5 mM MgCl_2_, and 100 μM MnCl_2_ (exceptionally, 50 µM MnCl_2_ was used to reduce the background of Polε activity on nicked M13 ssDNA). The indicated proteins were sequentially added: PrimPol (200 nM), Polε (10 nM), and RPA (20, 100, 500 nM). After 30 min at 37 °C reactions were stopped by adding Tris-EDTA buffer and purified with a Sephadex G-50 plus in 0.1% SDS to recover only labeled DNA. Samples eluted from the G-50 were dried and re-suspended in 20 µL of H_2_O. Loading buffer was added and reactions were analyzed in a 0.8% agarose gel. After electrophoresis, the gel was dried and analyzed by autoradiography.

## Results

### RPA stimulates the polymerase activity of PrimPol on long ssDNA templates

**Figure 1:**
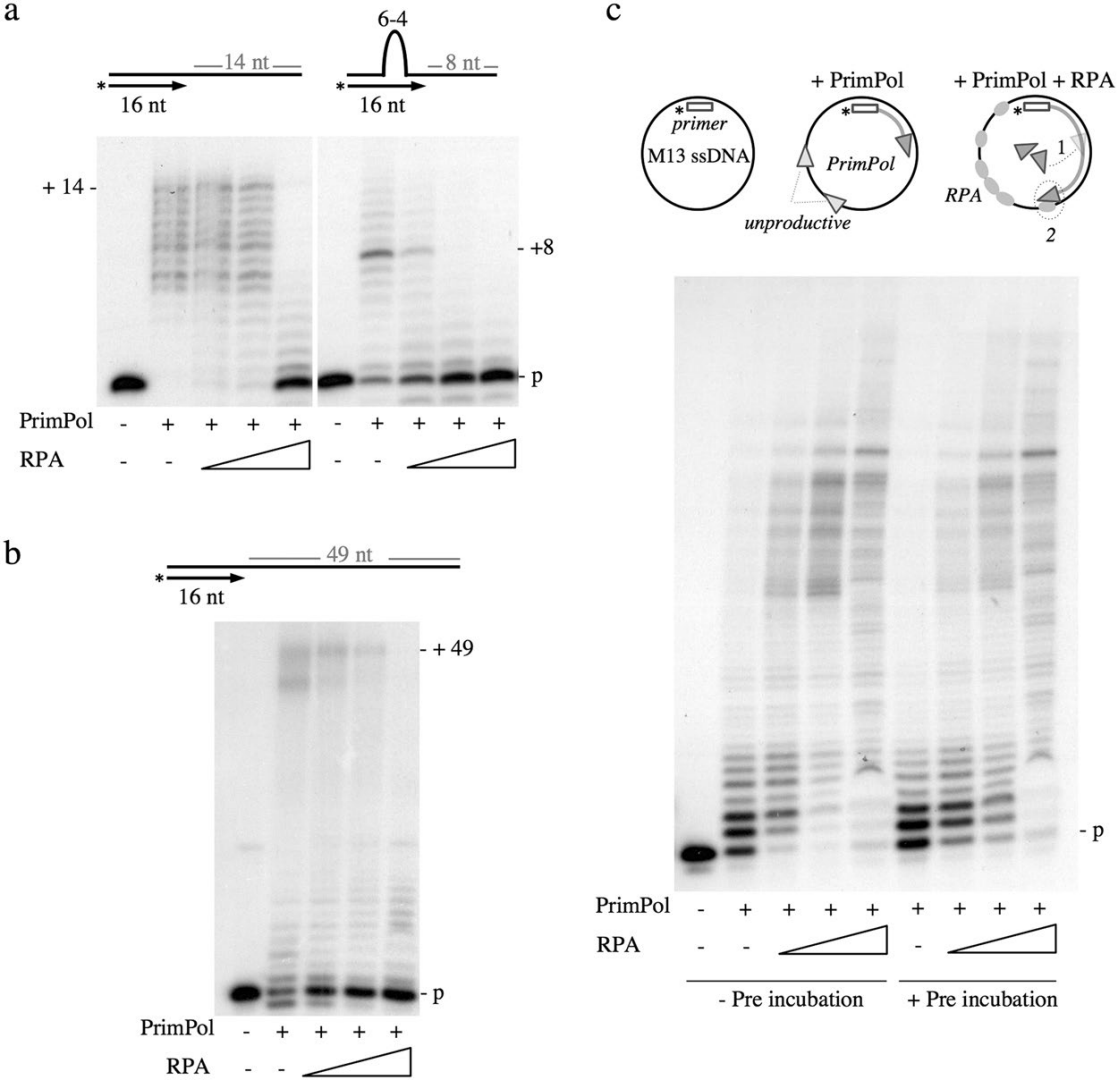
Modulation of PrimPol polymerase activity by RPA. (**a**) Effect of RPA on PrimPol polymerase activity on a short template/primer structure (30-mer/16-mer), either with an undamaged control template (left panel) or with a template containing a thymine dimer 6-4 photoproduct (right panel). PrimPol (200 nM) was incubated either alone or with RPA (12, 50 and 200 nM) in buffer R supplemented with 100 µM dNTPs and 2 nM labeled DNA. (**b**) Effect of RPA on PrimPol polymerase activity on a longer template/primer structure (65-mer/16-mer). PrimPol (200 nM) was incubated either alone or with RPA (12, 50 and 200 nM) in buffer R supplemented with 100 µM dNTPs and 2 nM labeled DNA. (**c**) Effect of RPA on PrimPol polymerase activity on circular M13 ssDNA annealed to a labeled oligonucleotide. PrimPol (200 nM) and RPA (20, 100, 500 nM) when indicated, were incubated with dNTPs (100 µM), 5 mM MgCl_2_ and 1 mM MnCl_2_ as metal cofactors, and primed-M13 ssDNA (2.5 nM). When indicated, PrimPol and RPA were pre-incubated 10 min at RT before template/primer addition. This experiment has been repeated more than 3 times, showing the same effect in all cases. The scheme represents the stimulation of PrimPol polymerase activity when increasing concentrations of RPA are added to the reaction when ssDNA is covered by RPA, PrimPol molecules cannot be unproductively bound, but form a recycling pool to fuel binding and extension of the primer (1); besides, a specific PrimPol/RPA association (2) can directly enhance the rate and processivity of polymerization.

First, we evaluated the effect of RPA on the extension of a 5′ labelled primer hybridized either to an undamaged template (Fig. 1a, left), or to a damage template with a 6-4 photoproduct thymine dimer (6-4PP) (Fig. 1a, right). In both cases, primer extension by PrimPol was inhibited when RPA was provided at increasing concentration. Inhibition in the control template was only observed when providing an equimolar concentration of PrimPol and RPA, suggesting that in such a short undamaged ssDNA PrimPol and RPA have similar binding capacity. Interestingly, this inhibition was more severe when the template contained a 6-4 PP lesion (Fig. 1a, right). It has been described that RPA recognizes and binds lesions in the DNA^19^, and this could be enhancing its binding capacity and subsequent inhibition of primer extension by PrimPol. Another explanation is that even the lowest concentration of RPA used is enough to prevent primer realignment by PrimPol as a way to skip the 6-4PP lesion^13^ (see the scheme at Fig. 1a, right) because RPA occupies the template. We next tested the extension of a similar primer but annealed to a longer oligonucleotide (65-mer), thus providing a ssDNA template of 49 nt. A similar inhibition of PrimPol polymerase activity by RPA was observed also in this substrate, suggesting that both proteins are competing for this relatively short template ssDNA (Fig. 1b). In summary, RPA inhibited polymerase activity of human PrimPol, confirming the results published by Guilliam and coworkers^16^. In our study, the inhibitory effect of RPA on PrimPol activity was enhanced, as higher concentrations of RPA were present in the reaction. RPA shows a high affinity for ssDNA, having a ssDNA binding site of approximately 30 nucleotides^20^. Thus, it is likely that RPA binds the template strands used in our polymerases assays (14 and 49 nt, respectively) with a higher affinity than PrimPol. It is also possible that the unwinding activity of RPA melts the primer, precluding PrimPol extension.

Next we used M13 ssDNA as a long template (7 kb) hybridized to a labeled primer, to provide PrimPol and RPA with longer ssDNA tracks to either compete or cooperate in binding. This substrate, mostly single-stranded, exaggerates replicative stress conditions, i.e. accumulation of long stretches of ssDNA due to the uncoupling of the replicative polymerase and the helicase at the replication fork. When provided alone, PrimPol can bind the 3′end of the labeled primer (in addition to be unproductively bound to multiple sites of the template), thus catalyzing its elongation (Fig. 1c). When RPA was added to the reaction, there was an increase in the amount and size of PrimPol elongation products, especially in the presence of the highest concentration of RPA (500 nM) that could potentially cover all M13 ssDNA. When PrimPol and RPA were pre-incubated before adding the DNA substrate, no differences in the pattern of elongation were observed (Fig. 1c), suggesting that a potential interaction of these two proteins is very effective and occurs in a very short time lapse, or requires their interaction with DNA. On the other hand, pre-incubation of RPA with primed-M13 ssDNA before PrimPol addition reduced the stimulatory effect of RPA, but even at highest RPA concentration used, PrimPol was able to extend the primer as efficiently as in the absence of RPA (Supplemental Fig. 2).

Therefore, the results obtained with a long ssDNA template (Fig. 1c) contrast with those shown before in small DNA template/primer molecules (Fig. 1a and b). The enhancement of PrimPol polymerase activity by RPA on a long ssDNA template may be due to different reasons: i) in this long ssDNA template, and at any concentration used, RPA does not compete with PrimPol for binding the primer/template junction, resulting in no inhibition of primer extension. ii) RPA can stabilize ssDNA ahead of PrimPol, removing secondary structures and allowing PrimPol to be more processive; iii) PrimPol is able to displace RPA from ssDNA when moving along the DNA during primer elongation; iv) if ssDNA is covered by RPA, PrimPol molecules that could be unproductively bound to the template are now available either for initial binding to the primer-terminus, or for recycling during primer extension, thus improving both the amount and length of the elongation products. v) It cannot be discarded that a specific association of PrimPol and RPA could be directly affecting the elongation rate and/or processivity of PrimPol.

### RPA stimulates the primase activity of PrimPol on long ssDNA templates

**Figure 2:**
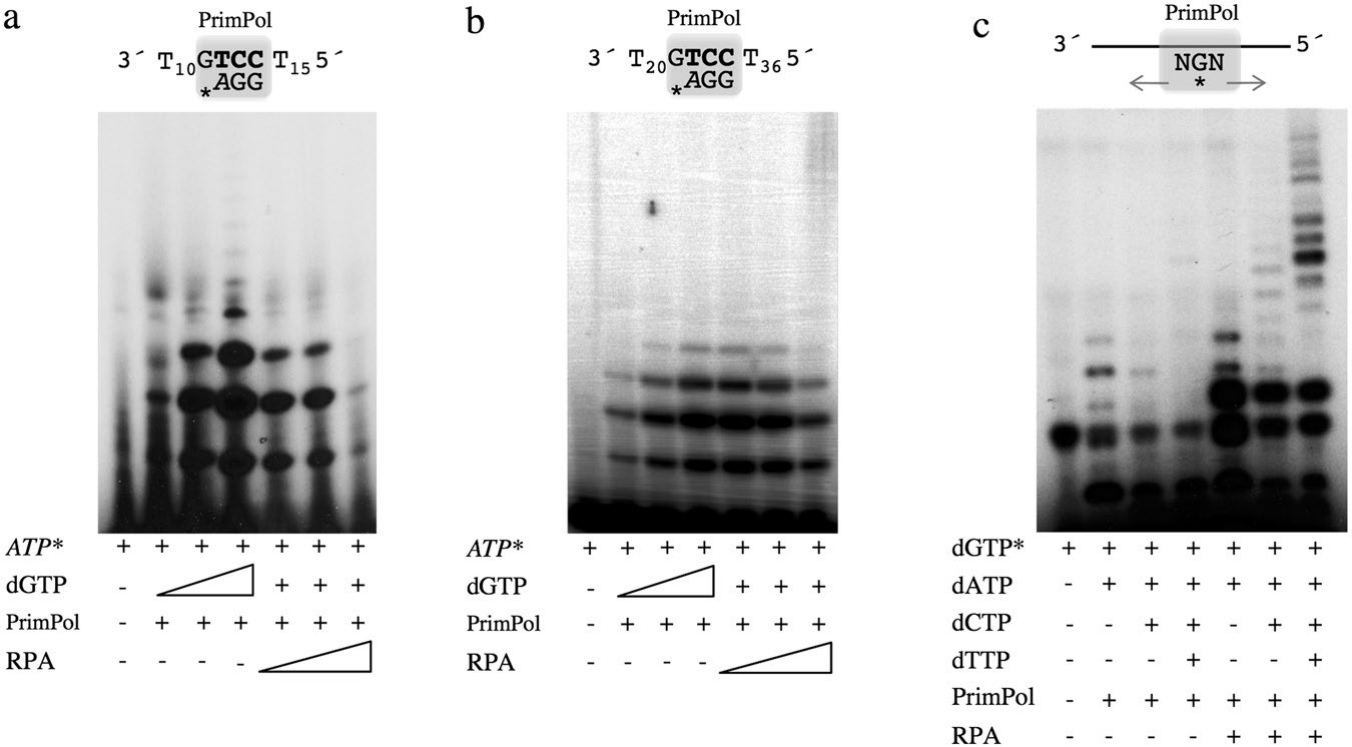
Modulation of PrimPol primase activity by RPA. The inhibitory effect of RPA on PrimPol primase activity was evaluated using two preferred templates differing in their size: (**a**) 29-mer GTCC or (**b**) 60-mer GTCC. The primase reaction contained 16 nM [γ-^32^P]ATP, dGTP (1, 10, 100 µM), 5 mM MgCl_2_ and 1 mM MnCl_2_ as metal cofactors, 1 µM of the indicated template, PrimPol (200 nM), and RPA (12, 50, 200 nM) when indicated. (**c**) RPA stimulates PrimPol primase activity on a heterogeneous 65-mer oligonucleotide. The reaction contained t16 nM [α-^32^P]dGTP, 100 µM of dATP, dCTP and dTTP as indicated, 5 mM MgCl_2_ and 1 mM MnCl_2_ as metal cofactors, PrimPol (200 nM), and RPA (200 nM) when indicated. The schemes depict the template oligonucleotide, PrimPol (grey square), and the synthesized primer product.

The effect of RPA on the specific primase activity of human PrimPol was evaluated by using a short (29-mer) ssDNA template that contains a preferred priming site (GTCC), as described^7^. As shown in Fig. 2a, PrimPol made short primers (2 to 4-mer) when using [γ-^32^P]ATP as the 5′ nucleotide and dGTP as the 3′ nucleotide, starting at the TC templating bases. When increasing concentrations of RPA (12.5, 50, 200 nM) were added to the optimised reaction of PrimPol with 100 µM dGTP, we observed a reduction in the amount of newly synthetized primers even at the lowest concentration of RPA (a stoichiometry of 1:10 RPA:PrimPol molecules). The same primase reaction was evaluated by using a longer ssDNA (60-mer) ssDNA template that also contains the preferred (GTCC) priming site (Fig. 2b). In this case, inhibition by RPA in this longer template was only observed at the highest concentration used. As demonstrated by EMSA, PrimPol binds this 60-mer GTCC favourite substrate with a similar affinity than RPA (see Supplemental Fig. 3). To study the effect of RPA on a more physiological template sequence, PrimPol primase activity was evaluated using a heterogeneous 65-mer oligonucleotide (Fig. 2c). PrimPol alone synthesized short primers on this substrate, even in the presence of the 4 dNTPs. Interestingly, addition of RPA produced a significant increase in the amount and size of the primers made by PrimPol (Fig. 2c). Thus, PrimPol primase activity in this relatively long ssDNA template was enhanced by RPA, unlike the inhibitory effect shown in the other two templates. It is important to consider that priming by PrimPol in the first two specific templates requires positioning of PrimPol in the central region of the template, whereas PrimPol could occupy more distal positions to initiate synthesis in the non-specific 65-mer template.

### PrimPol binds ssDNA in the presence of RPA and increases the affinity of RPA for ssDNA

**Figure 3:**
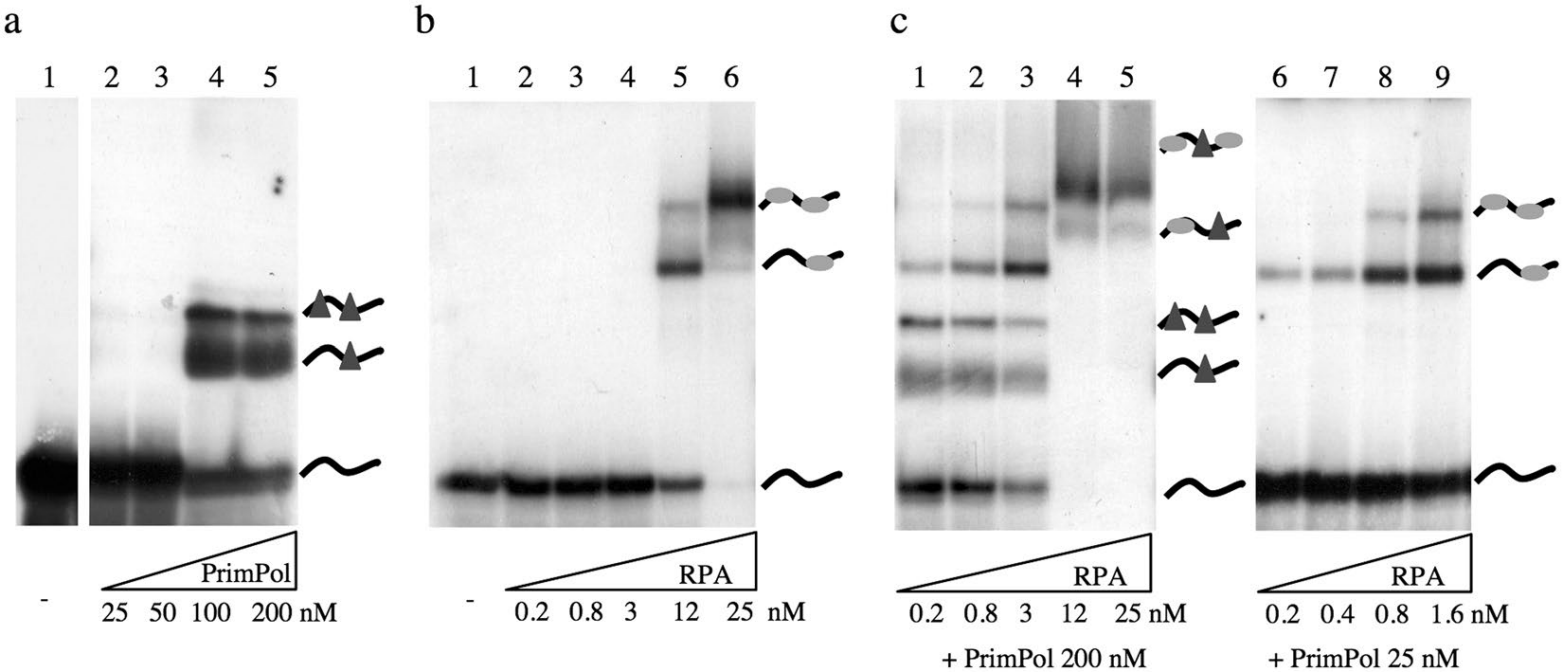
Mutual interaction of PrimPol and RPA with ssDNA. EMSA carried out as described in Materials and Methods, in the presence of a 65-mer ssDNA (2 nM) and the indicated concentration of PrimPol (**a**), RPA (**b**), or a combination of both proteins (**c**). The different retarded bands, representing individual protein:ssDNA complexes, were resolved by electrophoresis and autoradiography.

We investigated if the effect of RPA on PrimPol activities could be due to a modulation of their DNA binding capacity. For that, we performed Electrophoretic Mobility Shift Assays (EMSA) using PrimPol and RPA, separately or combined, and the labelled ssDNA template of 65-mer previously used in the primase assay (Fig. 2c). As shown in Fig. 3, each individual protein was able to bind this oligonucleotide, although with different affinity. The concentration of PrimPol required to produce a shift in the mobility of the labelled 65-mer was about 8–10 times higher than that of RPA (compare Fig. 3a and b), reflecting a higher affinity of RPA for this ssDNA. It is worth noting that RPA showed a similar binding affinity for this 65-mer oligo and for the 60-mer GTCC; conversely, PrimPol affinity for the 65-mer oligo was much lower than that displayed on the 60-mer GTCC favourite template (see Supplemental Fig. 3).

As shown in Fig. 3a, PrimPol produces two different retarded bands appearing at 100–200 nM (lanes 4 and 5), that likely represent one or two PrimPol molecules bound to the ssDNA template, or alternatively, the association of two PrimPol:ssDNA (1:1) complexes. RPA also produced two different retarded bands (observed at 12 nM and 25 nM; Fig. 3b, lanes 5 and 6), interpreted in this case as ssDNA associated with either one or two molecules of RPA. This interpretation agrees with the length of the template used (65-mer), and with a ssDNA binding site for RPA reported to span approximately 30 nt^20^. Interestingly, when PrimPol (200 nM) and RPA (12–25 nM) were simultaneously provided, super-shifted bands now appeared indicating the formation of hybrid complexes of ssDNA and the two proteins (Fig. 3c, lanes 4 and 5). As shown before, RPA concentration below 3 nM was not enough to produce a stable RPA:ssDNA complex by EMSA (Fig. 3b, lanes 2–4); however, the RPA-specific complexes were readily observed at low RPA concentration if PrimPol was provided at 200 nM (Fig. 3c, lanes 1–3). Thus, at low RPA and high PrimPol concentration there is a mix of the individual binding pattern of each protein (lanes 1–3), indicating that RPA is able to join and retard ssDNA more efficiently (about 4-fold better) than in the absence of PrimPol (compare Fig. 3b lane 5 with Fig. 3c lane 3). Strikingly, these RPA:ssDNA complexes were also observed when combining a lower concentration of PrimPol (25 nM), and low concentrations of RPA (0–2 to 1.6 nM), insufficient to retard/bind ssDNA when each protein was added individually. In these conditions, 12-fold less RPA added in combination with PrimPol was needed to give the same pattern of retarded bands than RPA alone (compare Fig. 3b lane 5 with Fig. 3c lane 8). These experiments demonstrate that both proteins can simultaneously bind ssDNA when provided at the adequate concentration, and suggest the existence of a specific interaction between PrimPol and RPA that increases the avidity/stability of the RPA:ssDNA interaction.

### RPA stimulates both the primase and polymerase activities of PrimPol in synergy with Polε

**Figure 4:**
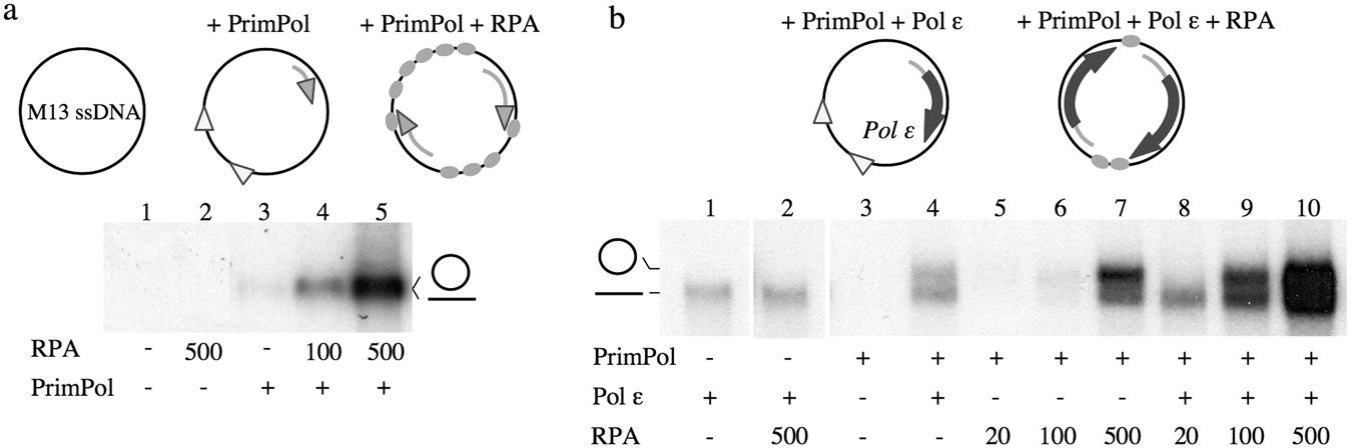
RPA stimulates both primase and polymerase activities of PrimPol in synergy with Polε. (**a**) Effect of RPA on the combined primase and polymerase activities of PrimPol in non-primed M13 ssDNA. PrimPol (200 nM) was incubated with the template M13 ssDNA 2 nM, 100 µM of dATP, dCTP, dTTP and GTP, 16 nM [α-^32^P] dGTP in the presence of 5 mM MgCl_2_ and 100 µM MnCl_2_ as metal cofactors; when indicated, RPA was added to the reaction at a concentration of 100 or 500 nM. (**b**) PrimPol-Polε combined assay was carried out essentially as described in part (**a**) but using 50 µM MnCl_2_, and when indicated adding Polε (10 nM) and RPA (20, 100 or 500 nM). After native agarose electrophoresis, the nature of the labelled products (circular ssDNA and linear ssDNA) was inferred from EtBr-staining of the gels prior to autoradiography. The schemes represent the stimulation of PrimPol primase and polymerase activities in the presence of RPA, and the subsequent stimulation on extension by Polε.

To gain further insights in the stimulatory effect of RPA, we measured both primase and polymerase activities of PrimPol using M13 ssDNA as template, without providing any pre-existing primer, and using limiting Mn^2+^ (100 µM) and PrimPol concentration. Under these conditions (Fig. 4a) PrimPol was rather inefficient but able to synthesize *de novo* primers which were further extended (labelled by incorporation of [α-^32^P]dGTP) and analysed in native agarose gels (Fig. 4a lane 3). Strikingly, the intensity of the labelled band dramatically increased when RPA was provided at a concentration either partially (lane 4) or fully (lane 5) covering M13 ssDNA. This result is explained by the stimulatory effect of RPA on this template, not just on the polymerase activity of PrimPol but also facilitating or at least allowing its primase activity.

Figure 4b shows a similar experiment as described before but now also providing the leading strand replicative polymerase, Polε, to extend the primers made by PrimPol, thus mimicking the rescue, via repriming, of a stalled leading strand^9^. When Polε was used alone (lane 1), there was a labelled band running faster than M13 circular-ssDNA, at the position of linear (nicked) M13 ssDNA, likely due to some unusual nucleotide incorporation at the 3′-ends; this product was not affected by the presence of RPA (lane 2). PrimPol alone (lane 3) at even lower Mn^2+^ concentration (50 µM) does create *de novo* primers that are only evidenced when extended by Polε, as they run with the circular (intact) template under native conditions (lane 4, upper band). In agreement with the experiment shown in Fig. 4a, increasing concentrations of RPA (lanes 5–7) significantly increased these specific PrimPol products, again implying a stimulatory effect of RPA on both primase and polymerase activities of PrimPol. Importantly, this stimulation by RPA was also observed when the reaction was enhanced by the presence of both PrimPol and Polε (lanes 8–10).

Collectively, these new findings supports the existence of a functional complex between PrimPol and RPA that allows repriming at the exposed ssDNA regions formed in the leading strand upon replicase stalling.

### Concluding remarks

**Figure 5:**
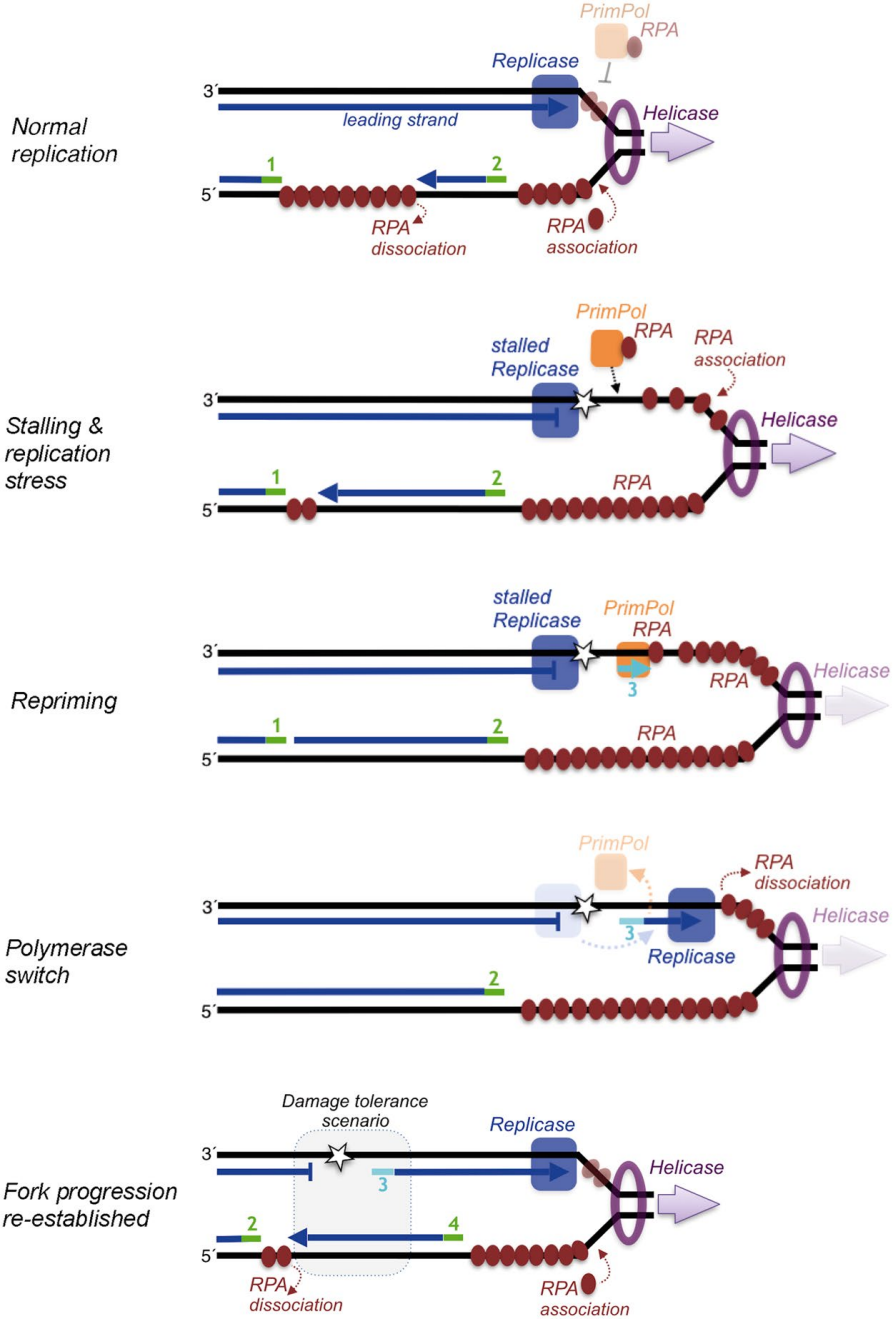
Model for PrimPol and RPA interactions at the replication fork during replicative stress. During normal replication fork progression, the leading replicase and the helicase are coordinated, RPA mainly binds to the lagging strand and PrimPol (depicted in association to RPA) has no access to the short ssDNA ahead of the leading replicase (likely covered by RPA). Under replicative stress, the leading strand replicase is stalled, and PrimPol/RPA now can gain access to the long stretches of ssDNA accumulated as a consequence of a sustained helicase unwinding, in a way compatible with an improved binding of RPA to ssDNA. PrimPol repriming in the leading strand triggers a polymerase switch that mobilizes the leading strand replicase from the stalling site to the new DNA primer, that becomes elongated, and the excess of RPA becoming displaced and dissociated. Recovery of the coordination between replicase and helicase re-establishes fork progression and normal lagging strand synthesis. The gap left behind in the leading strand constitutes a damage tolerance scenario, now accessible to translesion and/or repair machineries. For simplicity, the primase and polymerases acting on the lagging strand are not represented. RNA primers in the lagging strand (green) or an eventual DNA primer made by PrimPol in the leading strand (cyan) are numbered according to their proposed order of synthesis.

During replication helicases are ahead of the replication fork, opening the double helix and delivering ssDNA template strands. Immediately behind, replicative polymerases catalyze the addition of complementary nucleotides to form the newly synthesized DNA strands, either continuously (leading strand) or in multiple steps that require new priming events at the lagging strand (Fig. 5). In such normal conditions, the length of ssDNA template in the leading strand is maintained relatively short and perhaps fully covered by RPA, thereby preventing PrimPol repriming on this ssDNA. The inhibition of PrimPol activities by RPA on short templates shown here, and in a previous report^16^, supports this hypothesis. On the other hand, a large RPA filament must be formed to prevent damage in the large “to be copied” template portion (Okazaki fragment) of the lagging strand, meanwhile a new primer is synthesized; again, PrimPol action is likely precluded as the RPA filament in the lagging strand is growing by adding RPA monomers cooperatively and in coordination with helix opening.

However, when there is a lesion in the DNA template or any other condition impeding elongation of the leading strand, the polymerase responsible could be blocked, becoming uncoupled to the unwinding helicase, thus promoting an accumulation of a long stretch of uncopied ssDNA template that could not be readily covered by RPA. Consequently, PrimPol finds the opportunity to bind in proximity to the stalling site, probably by an specific interaction with RPA that reinforces its avidity for ssDNA, synthesizing a new primer to restart leading strand synthesis and re-establishing normal fork progression (Fig. 5). The RPA-mediated stimulation of PrimPol activities shown here to occur with long ssDNA templates supports this model and resolves the apparent paradox of RPA being both a recruiting factor for PrimPol^6^ and an inhibitor of its primase and polymerase activities^16^. Importantly, this RPA-regulated repriming by PrimPol facilitates clearance of the replicase from the stalling site, preparing a separate and accessible scenario (a short gap) for damage tolerance that can occur either by direct reading of the lesion, or by template switch mediated by a recently synthesized Okazaki fragment (Fig. 5). This working model agrees with recent findings demonstrating that most of the translesion synthesis events occur after leading-strand repriming^21^.

## Electronic supplementary material

Supplementary information
