# Supplementary material for: Human PrimPol activity is enhanced by RPA
Source: Sci Rep. 2017 Apr 10;7:783. doi: 10.1038/s41598-017-00958-3 (PMC5429719; doi:10.1038/s41598-017-00958-3)

Human PrimPol activity is enhanced by RPA

Supplemental information

María I. Martínez-Jiménez[#], Antonio Lahera[#], Luis Blanco*

Centro de Biología Molecular Severo Ochoa (CSIC-UAM), c/ Nicolás Cabrera 1, 28049 Cantoblanco, Madrid, Spain.

Supplemental information Figure 1. Purified human PrimPol and human RPA proteins used in this work

The purity of the final fractions, obtained as indicated in Materials and Methods, was analysed by 15% SDS- polyacrylamide gel electrophoresis followed by staining with colloidal Coomassie G-250. Lane 1: unstained protein standard, broad range (New England Biolabs); lane 2: purified human PrimPol (0,7 μg), having a theoretical molecular weight of 67 kDa (including the N-terminal 10xHis tag and the linker peptide), has an electrophoretic mobility that is slightly lower than expected (as described in García-Gómez *et al.*, 2013),); lane 3: purified heterotrimer of RPA (1 μg), composed of subunits RPA 1 of 70 kDa RPA 2 of 32 kDa and RPA 3 of 14 kDa, purified as described in Binz *et al.*, 2006 (17).

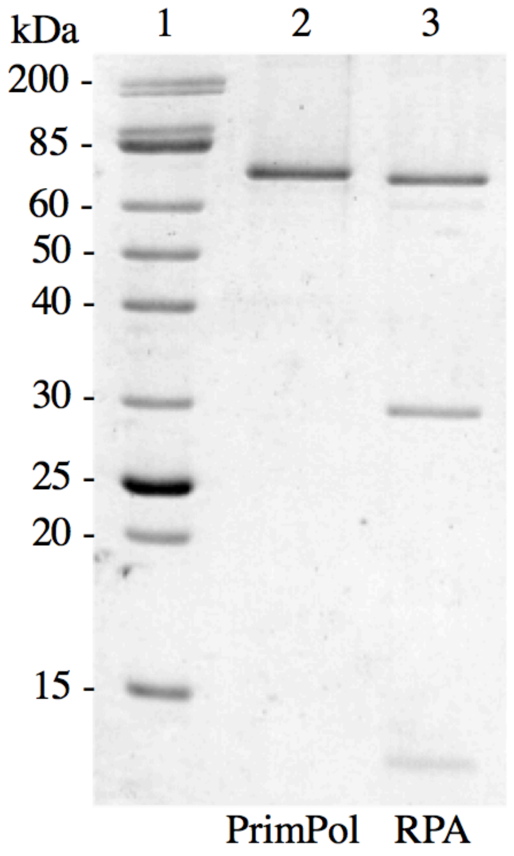

Supplemental information Figure 2. Pre-incubation of RPA with primed-M13ssDNA before PrimPol addition reduces the stimulatory effect of RPA on PrimPol polymerase activity

Reactions (in 20 μL) were carried out in a buffer containing: 50 mM Tris-HCl [pH 7.5], 50 mM NaCl, 100 μM $MnCl_2$, 5 mM $MgCl_2$, 1 mM DTT, 2.5% glycerol, 0.1 mg/ml BSA, 100 μM dNTPs and 2.5 nM 5′ [γ-^{32}P]-labelled-primer (5'-GTTTTCCAGTCACGAC-3') annealed to M13ssDNA supplied by New England Biolabs (Ipswich, MA, USA). Left panel: PrimPol 100 nM and when indicated RPA (20, 100 and 500 nM) were added simultaneously to the reaction and incubated during 10 min at 37ºC. Right panel: When indicated, RPA (20, 100 and 500 nM) was pre-incubated with all the components of the reaction (with the exception of PrimPol) for 10 min at RT, and then PrimPol was added at 100 nM and the incubation was continued during 10 min at 37 ºC. Reactions were stopped by adding 8 μL of loading buffer, then heated 5 min at 80ºC and analysed in a 15% polyacrylamide sequencing gels containing 8 M urea.

As expected, pre-incubation with increasing concentrations of RPA progressively reduced the stimulation observed when both PrimPol and RPA are simultaneously added. This inhibition could be in part due to unwinding of the primer by RPA, in addition to a favoured competition for the ssDNA portion of the template strand. However, and even using the highest RPA concentration at the pre-incubation step, PrimPol was able to extend the primer at a similar efficiency than that obtained in the total absence of RPA. These results suggest that for optimal PrimPol and RPA functional interaction, PrimPol must be promptly recruited to the accumulated ssDNA.

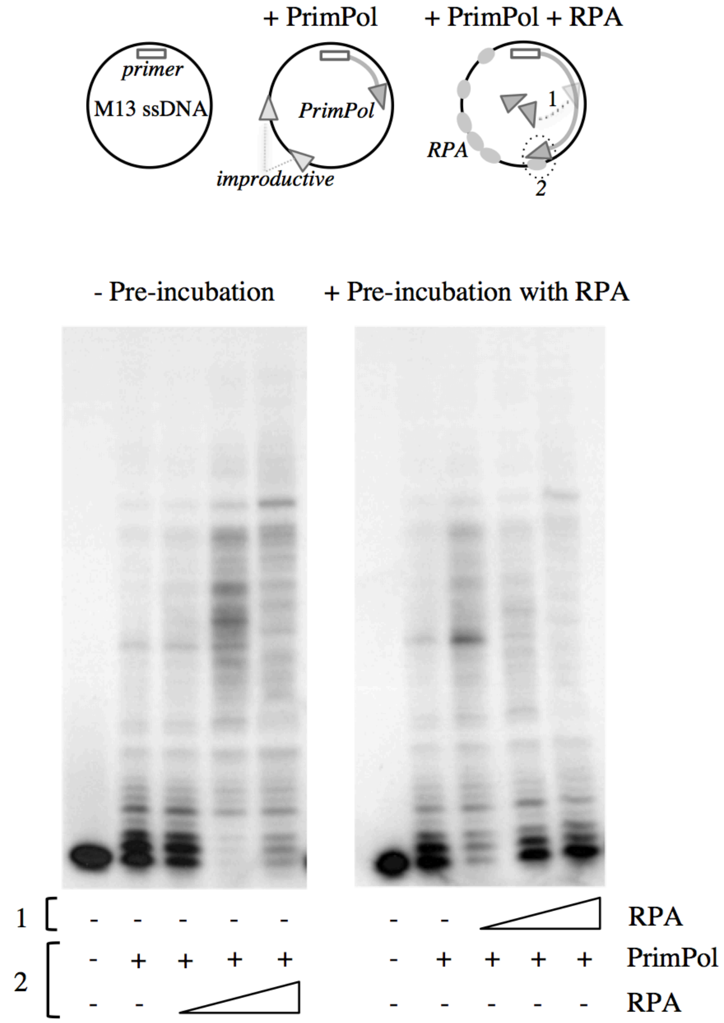

Supplemental information Figure 3. PrimPol and RPA showed similar binding affinity for a T-rich ssDNA oligonucleotide containing a favourite PrimPol priming site (GTCC)

The binding affinity of PrimPol and RPA for a ssDNA substrate were compared by EMSA using the specific 60-mer GTCC, a favourite ssDNA substrate for PrimPol (see the scheme). EMSA was carried out in buffer containing: 50 mM Tris-HCl [pH 7.5], 50 mM NaCl, 1 mM $MnCl_2$, 1 mM DTT, 2.5% glycerol, 0.1 mg/ml BSA and 2.5 nM polyethylenglycol 4000. Increasing concentrations of PrimPol or RPA (1.5, 3, 6, 12 nM) were incubated with 2.5 nM of 5´[γ-^{32}P]-labelled 60-mer "GTCC" oligonucleotide during 20 min at 25ºC. The reaction was loaded in a 6% acrycrilamide gel in buffer 1x Tris-Gly and run at 180 V during 2h at 4ºC. The gel was dried and exposed to autoradiography. PrimPol and RPA displayed a similar binding affinity for the 60-mer GTCC oligonucleotide.

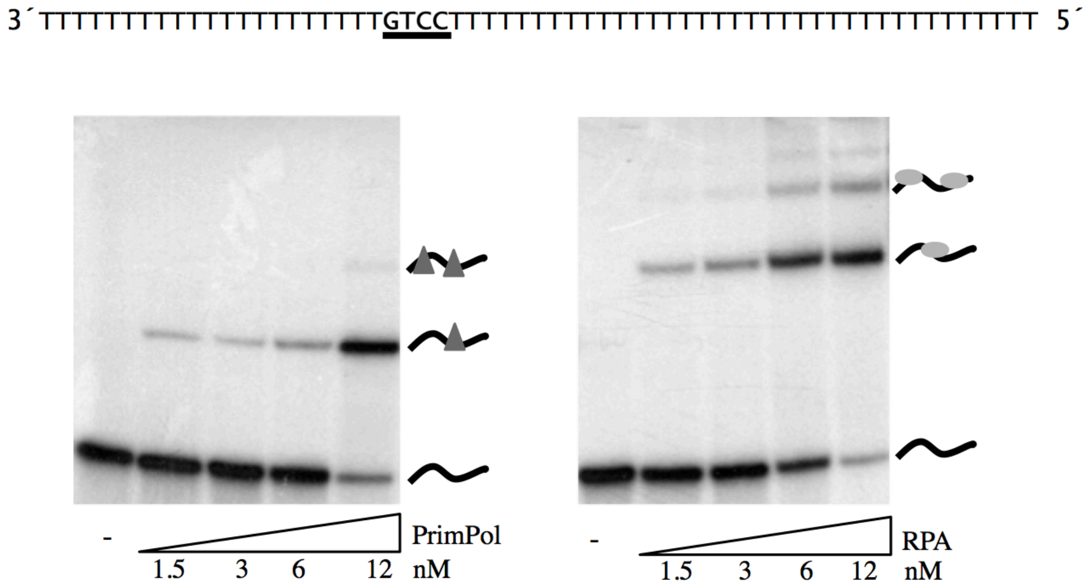

Supplement: Supplementary file 1 — Supplementary information [file 41598_2017_958_MOESM1_ESM.pdf]
